# Comparative Analysis of the Gut Microbiota in People with Different Levels of Ginsenoside Rb1 Degradation to Compound K

**DOI:** 10.1371/journal.pone.0062409

**Published:** 2013-04-29

**Authors:** Kyung-Ah Kim, IL-Hoon Jung, Se-Hoon Park, Young-Tae Ahn, Chul-Sung Huh, Dong-Hyun Kim

**Affiliations:** 1 Department of Life and Nanopharmaceutical Sciences and Department of Pharmacy, Kyung Hee University, Seoul, Korea; 2 R &B D Center, Korea Yakult Co., Ltd., Yongin-si, Kyunggi-do, Korea; University College London, United Kingdom

## Abstract

*Panax ginseng* (family Araliaceae) which contains ginsenoside Rb1 as a main constituent is traditionally used as a remedy for cancer, inflammation, stress, and ageing. The ginsenoside Rb1 in orally administered ginseng is metabolized to bioactive compounds by gut microbiota before their absorptions to the blood. However, its metabolizing activities in individuals are significantly different as we previously demonstrated. Here, we selected 5 samples with fecal activity potently metabolizing ginsenoside Rb1 to compound K (FPG; metabolic activity, 0.058±0.029 pmol/min/mg) and 5 samples with fecal activity non-metabolizing ginsenoside Rb1 to compound K (FNG) from a pool of 100 subjects investigated in a previous study and analyzed fecal microbiota by 16S rRNA gene pyrosequencing. Taxonomy-based analysis showed that the population levels of *Firmicutes* and *Proteobacteria* in FPG were lower than in FNG, but those of *Bacteroidetes* and *Tenericutes* in FPG were higher than in FNG. At the genus level, the population levels of *Clostridiales_uc_g, Oscillibacter, Ruminococcus, Holdemania,* and *Sutterella* in FPG were significantly higher than in FNG, but that of *Leuconostoc* in FPG was lower than in FNG. The population levels of *Bacteroides* and *Bifidobacterium,* which potently metabolizes ginsenoside Rb1 to compound K were dramatically increased in FPG. The gut microbiota compositions of FPG and FNG were segregated on PCO2 by Principal Coordinate Analysis. Intestinal bacterial metabolism of ginseng, particularly ginsenoside Rb1, may be dependent on the composition of gut microbiota, such as *Ruminococcus* spp., *Bacteroides* spp. and *Bifidobacterium* spp.

## Introduction

Most of traditional Chinese medicines (TCM) are orally administered to humans. The components of orally administered TCM are therefore inevitably brought into contact with intestinal microbiota in the alimentary tract [1], [2]. In human, gut is trillions of individual microbes residing [3], [4]. The exact membership of this highly complex gut ecosystem, known as the microbiome, varies between individuals. Integral to this picture is the interplay between gut bacteria and diet as well as between gut microbes and health [4], [5], [6]. The global rise of diets, such as fat and chronic diseases, such as obesity and bowel disease, is increasingly being linked with perturbations in gut flora. The gut microbiota has the ability to metabolize drugs and other xenobiotics more extensively than any other part of the body [7], [8], [9]. Thus, gut microbiota may transform the constituents of orally administered TCM to bioactive compounds before they get absorbed from the gastrointestinal tract [2], [10], [11].

Ginseng (the root of *Panax ginseng* C.A. Meyer, Araliaceae), which contains ginsenosides as major constituents, is frequently used as a traditional medicine in Asian countries [2]. The ginsenosides have been reported to show various biological activities including anti-inflammatory activity [12] and anti-tumor effects [13]. To express the pharmacological actions of ginseng saponins, it is presumed that these ginsenosides must be metabolized by human intestinal microbes after being taken orally [14], [15]. Thus, ginsenosides Rb1, Rb2 and Rc are metabolized to 20-*O*-β-D-glucopyranosyl-20(*S*)-protopanaxadiol (compound K) by human intestinal microbes and absorbed into the blood [10], [11], [16], [17], [18]. The metabolized compound K exhibits the potent anti-tumor, anti-inflammatory, and anti-allergic actions more than ginsenoside Rb1 [14], [19], [20]. Therefore, compound K-forming intestinal microbes play the important role in expressing the pharmacological effects of ginseng. However, the metabolic activity of ginsenoside Rb1 to compound K is variable between individuals, although the activity is not different between males and females or between ages [21], [22]. Nevertheless, studies on the relationship between the metabolic activities of TCM constituents and human gut microbiota composition have not been performed.

In the present study, we selected 5 samples with fecal activity potently metabolizing ginsenoside Rb1 to compound K (FPG) and 5 samples with fecal activity non-metabolizing ginsenoside Rb1 to compound K (FNG) from a pool of 100 subjects investigated in a previous study [22] and analyzed fecal microbiota by 16S rRNA gene pyrosequencing.

## Materials and Methods

### Materials

p-Nitrophenyl-β-D-glucopyranoside was purchased from Sigm-Aldrich (St. Louis, MO). Ginsenoside Rb1 (purity, >92%) and compound K (purity, >95%) were isolated using the previously published method of Bae *et al*. [16], [20].

### Subjects

From a pool of 100 subjects analyzed in a previous study [22], we selected 5 samples with FPG (sample No. 3, 10, 29, 47, and 95; 38.0±13.3 years) and 5 samples with FNG (sample No. 20, 23, 31, 75, and 88; 38.4±12.7 years). Exclusion criteria included smoking and current medication, especially regular or current use of antibiotics. The recruitment of subjects and the consent procedure as well as the collection of their stools were approved by the ethics committee for the Care and Use of Clinical Study in the Medical School, Kyung Hee University (KMC IRB 0922-08-A1). The participants provided their written informed consent to participate in the study.

### Sample Preparation

The human fecal samples (about 1 g) were prepared according to the previous method [22], were collected in plastic cups 9 h after fasting, and then carefully mixed with a spatula and suspended with cold 9 ml saline. The fecal suspension was centrifuged at 500×g for 5 min. The supernatant was then centrifuged at 10,000×g for 20 min. The resulting precipitates were used as a metabolic enzyme source for the assay of enzyme activity. The preparation and assay of the enzyme source were performed within 24 h under anaerobic conditions.

### Assay of β-D-glucosidase, β-D-glucuronidase, β-D-galactosidase and α-L-rhamnosidase Activities

For the assay of β-D-glucosidase, β-D-glucuronidase, β-D-galactosidase, and α-L-rhamonosidase activities, the reaction mixture (total volume of 0.5 ml) was composed of 0.2 ml of 1 mM p-nitrophenyl-β-D-glucopyranoside, p-nitrophenyl-β-D-glucuronide, p-nitrophenyl-β-D-galactopyranoside, or p-nitrophenyl-α-L-rhamnopyranoside as substrate respectively, 0.2 ml of 0.1 M phosphate buffer (pH 7.0), and 0.1 ml of the fecal enzyme fraction. The reaction mixture was incubated at 37°C for 20 min. The reaction was stopped by the addition of 0.5 ml of 0.5 N NaOH, centrifuged at 3,000×g for 10 min and measured the absorbance at 405 nm (UV-vis spectrophotometer, Shimadzu UV-1201). The activities were expressed in pmol per minute per milligram protein and the protein content was assayed by Bradford method [23].

### Assay of Intestinal Bacterial Enzyme Activity Metabolizing Ginsenoside Rb1, and Ginseng Extract to Compound K

For the fecal enzyme activity for ginsenoside Rb1 or ginseng extract, the reaction mixture (0.5 ml) containing 0.125 ml of the human fecal suspension and 0.1 mM ginsenoside Rb1 (or 0.5 mg ginseng extract) was incubated at 37°C for 4 h, and 1.5 ml of MeOH was added to stop the reaction. The reaction mixture was centrifuged at 3,000×g for 10 min and the level of ginsenoside Rb1 in the resulting supernatant was analyzed by HPLC.

### HPLC Analysis

The reaction mixture was analyzed by Hewlett Packard Series 1050 HPLC system. The instrument was controlled and the data were processed by a HP Chemstation (Rev. A. 09.03). The analytical column was an Agilent Hypersil ODS (100×4.6 mm i.d., 5 µm; Agilent Technologies, USA) protected by a C18 Security Guard Cartridge (Phenomenex, Torrance, CA). The elution solvent was acentonitrile (ACN) and distilled and deionized water (DDW). Ginsenoside Rb1 was analyzed using a linear gradient 0∼70% ACN in DDW including 0.05% formic acid for 15 min and an isocratic elution for 5 min in 70% ACN at a flow rate of 1.0 ml/min and detected at 203 nm. A sample volume of 20 µl was used for injection. The retention times of Rb1, and compound K were 10.5, and 15.6 min, respectively.

### DNA Extraction, Pyrosequencing, and Data Analysis

Genomic DNA was extracted from fecal sample using a commercial DNA isolation kit (QIAamp DNA stool mini kit, Qiagen, Hilden, Germany) by following the manufacturer’s protocol. For pyrosequencing, amplification of genomic DNA was performed using barcoded primers, which targeted the V1 to V3 region of the bacterial 16S rRNA gene. The amplification and sequencing were performed according to the methods described by Chun et al. [24] and completed by Chunlab Inc. (Seoul, Korea) using a 454 GS FLX Titanium Sequencing System (Roche, Branford, CT). Sequence reads were identified using EzTaxon-e database (http://eztaxon-e.ezbiocloud.net/; [25]) on the basis of 16S rRNA sequence data. Number of sequence analyzed, observed diversity richness (operational taxonomic units, OTUs), estimated OTU richness (ACE and Chao1), and coverage in the present pyrosequencing were indicated in Table 1.

**Table 1:** Number of sequence analyzed, observed diversity richness (OTUs), estimated OTU richness (ACE and Chao1), and coverage.

			Phylotype			
Group	Number	Total reads	OTUs	ACE	Chao1	Goods Coverage
FPG	3	10320	1262	2183.3	1912.8	0.95
	10	4959	713	1374.7	1168.0	0.94
	29	9271	1039	2083.4	1624.8	0.95
	47	7108	271	330.7	336.0	0.99
	95	9536	1015	1933.3	1574.3	0.95
Mean±SD		8238±2186	860±382	1581±765	1323±612	0.96±0.02
FNG	20	12807	1293	1874.7	1913.3	0.96
	23	11353	436	740.8	631.0	0.99
	31	3851	419	706.1	596.2	0.96
	75	2453	249	492.6	414.1	0.96
	88	5077	300	484.5	413.2	0.98
Mean±SD		7108±4661	539±428	793±634	859±579	0.97±0.01

The cutoff value of phylotype is equal to or greater than 97% similarity. OTUs, operational taxonomic units; FPG, fecal activity potently metabolizing ginsenoside Rb1 to compound K; FNG, fecal activity non-metabolizing ginsenoside Rb1 to compound K.

### Statistics

The data are expressed as the means±standard deviation. Statistical analysis of the data was performed with Student’s *t*-test. Differences with a *p*<0.05 were considered to be statistically significant.

## Results and Discussion

**Figure 1:**
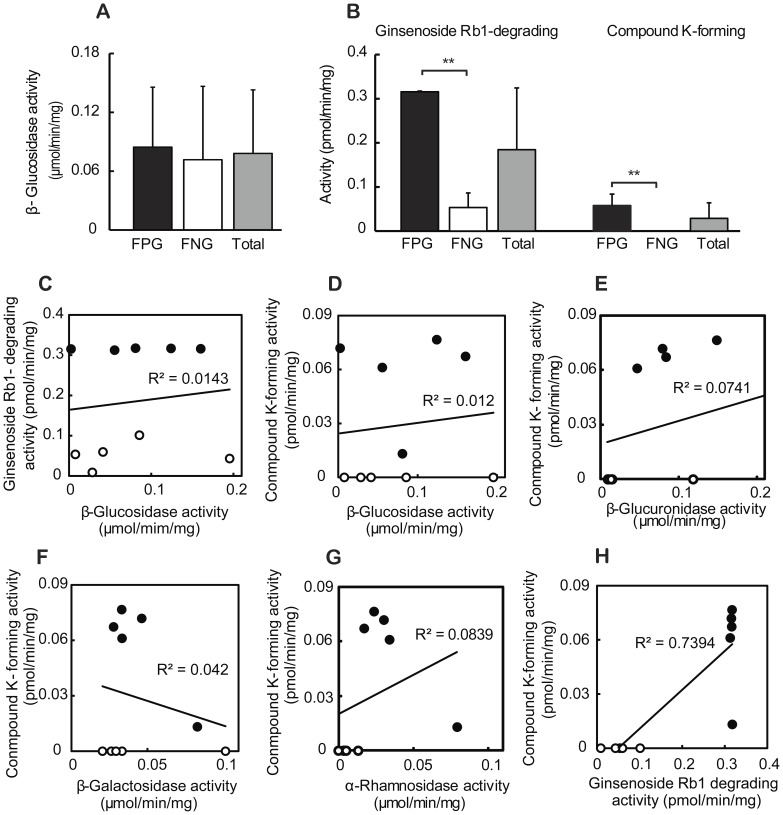
Fecal metabolic activities for p-nitrophenyl-β-D-glucopyranoside, p-nitrophenyl-β-D-glucuronide, p-nitrophenyl-β-D-galactopyranoside, p-nitrophenyl-α-L-rhamnopyranoside and ginsenoside Rb1 in 10 Koreans. (A) Hydrolytic activity of p-nitrophenyl-β-D-glucopyranoside (PNG). (B) Hydrolytic activitiy of ginsenoside Rb1 to compound K. The relationships between PNG hyfrolyzing and ginsenoside Rb1 degrading activities (C), between PNG hydrolyzing and compound K forming activities (D), β-D-glucuronide hydrolyzing and compound K forming activities (E), β-D-galactopyranoside hydrolyzing and compound K forming activities (F), α-L-rhamnopyranoside hydrolyzing and compound K forming activities (G), and between ginsenoside Rb1 degrading and compound K forming activities (H). We selected 5 samples with FPG (fecal activity potently metabolizing ginsenoside Rb1 to compound K) and 5 samples with FNG (fecal activity non-metabolizing ginsenoside Rb1 to compound K) from a pool of 100 subjects investigated in a previous study [22]. FPG is black bars (in A and B) and closed circles (in C, D and E). FNG is white bars (in A and B) and open circles (in C, D and E). Grayish bars (in A and B) are average values of 10 samples. All values indicate mean ± SD. **p<0.01.

The pharmacological activities of orally administrated herbal medical components such as ginsenoside Rb1 are enhanced by gut microbiota [16]. Furthermore, the capacities of transformation of bioactive compounds are variable between individuals. Therefore, to understand the difference of gut microbiota related to the fecal metabolism of ginsenoside Rb1 to compound K between individuals, we selected 10 samples from a pool of 100 subjects analyzed in a previous study [22]; 5 samples with FPG and 5 samples with FNG. The activity of the former group (FPG) potently metabolizing ginsenoside Rb1 to compound K was 0.058±0.029 pmol/min/mg whereas the latter group (FNG) did not metabolize ginsenoside Rb1 to compound K (Fig. 1B). However, using p-nitrophenyl-β-D-glucopyranoside as a substrate, β-glucosidase activity between FPG and FPG was not significantly different (Fig. 1A). Furthermore, their p-nitrophenyl-β-D-glucopyranoside-hydrolyzing β-glucosidase activities were not proportional to their ginsenoside Rb1 degrading activities or compound K-forming activities (Fig. 1C and D). In addition, the activities of β-glucuronidase hydrolyzing p-nitrophenyl-β-D-glucuronide, β-galactosidase hydrolyzing p-nitrophenyl-β-D-galactopyranoside, and α-rhamnosidase hydrolyzing p-nitrophenyl-α-L-rhamnopyranoside were not proportional to compound K-forming activities (Fig. 1E, 1F and 1G). However, ginsenoside Rb1-degrading activities were proportional to their compound K-forming activities (Fig. 1H).

**Figure 2:**
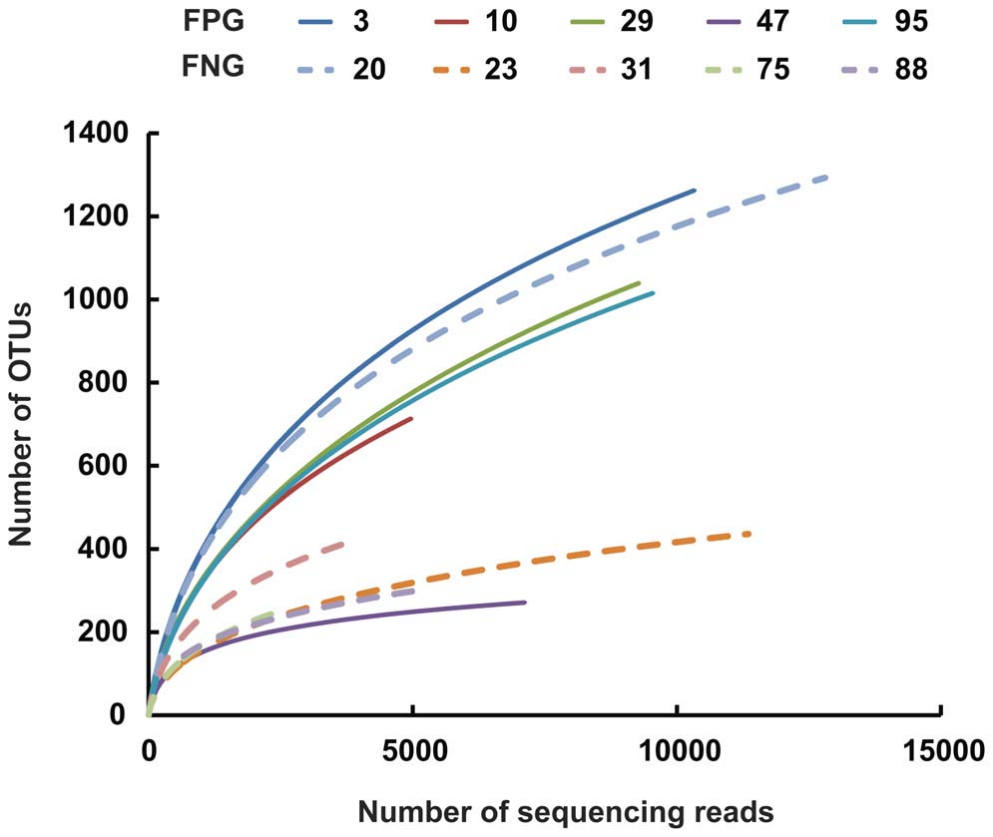
Rarefaction curves. Rarefaction analysis of V1–V3 pyrosequencing tags of the 16S rRNA gene in fecal microbiota from FPG (fecal activity potently metabolizing ginsenoside Rb1 to compound K) or FNG (fecal activity non-metabolizing ginsenoside Rb1 to compound K).

**Figure 3:**
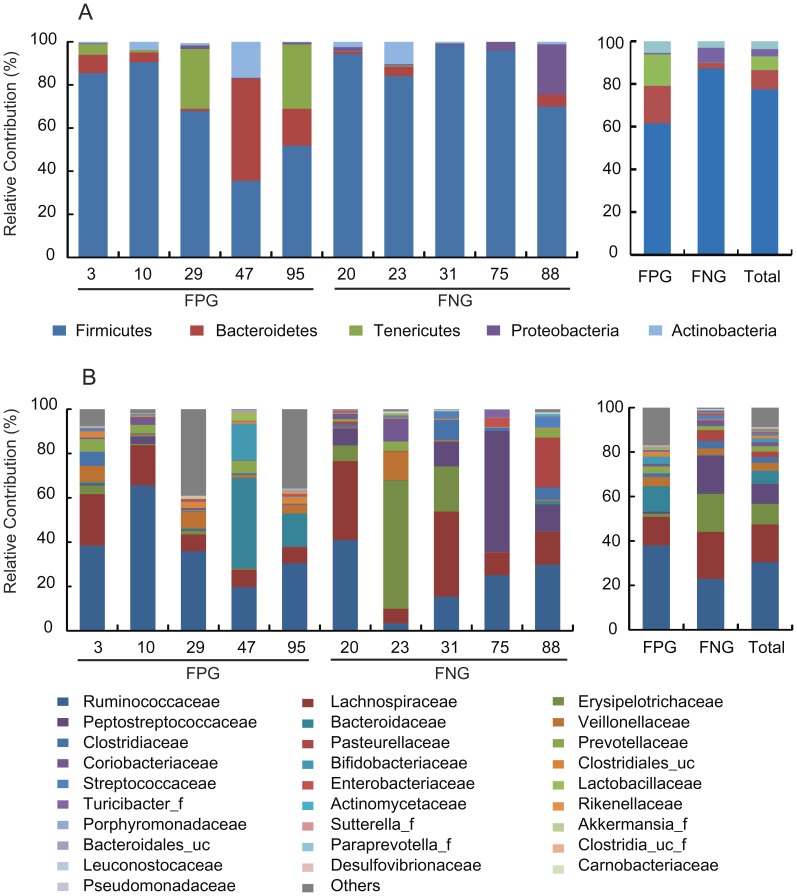
The composition of fecal microbiota in 10 Koreans. The relative contribution of dominant phyla (A) and families (B) identified from pyrosequencing data is shown (individual samples are on the left panels and pooled samples are on the right panels). FPG, fecal activity potently metabolizing ginsenoside Rb1 to compound K; FNG, fecal activity non-metabolizing ginsenoside Rb1 to compound K.

Next, we analyzed the gut microbiota compositions of FPG and FNG by pyrosequencing. As demonstrated by the rarefaction curves (Fig. 2) and the number of sequences analyzed and estimated OTU richness (Table 1), bacterial richness and diversity in FPG showed a tendency to be higher than in FNG with no significant difference. Furthermore, taxonomy-based analysis showed a modulation of the populations of the dominant intestinal microbiota. The distributions of the major phyla (*Firmicutes*, *Bacteroidetes*, Tenericutes, *Proteobacteria* and *Actinobacteria*) are consistent with previous human gut studies [26], [27], [28]. However, the main dominants in FPG were *Firmicutes*, *Bacteriodetes,* and *Tenericutes*, while those in FNG were *Firmicutes* and *Proteobacteria* (Figure 3A). Of them, the population levels of *Firmicutes* and *Proteobacteri*a in FPG were lower than in FNG, but those of *Bacteroidetes* and *Tenericutes* in FPG were higher than in FNG. At the family level, among the relative abundance of 16 major family groups, an average of 74.3% of all sequences belonged to the 8 families comprising Firmicutes: *Ruminococcaceae, Lachnospiraceae, Erysipelotrichaceae, Peptostreptococcaceae, Veillonellaceae, Clostridiaceae, Clostridiales_uc_g, and Streptococcaceae* (Fig. 3B). The two families of *Bacteroidetes* (*Bacteroidaceae and Prevotellaceae)* accounted for an average of 8.4% of sequences while other families (*Pasteurellaceae,* phylum *Proteobacteria; Coriobacteriaceae and Bifidobacteriaceae,* phylum *Actinobacteria)* accounted for an average of 5.9% of sequences. Interestingly, the population levels of *Ruminococcaceae, Bacteroidaceae, Sutterella_f, Clostridiales_uc*, *Bifidobacteriaceae,* and *Rikenellaceae* in FPG were higher than in FNG, while those of *Lachnospiraceae, Erysipelotrichaceae, Peptostreptococcaceae, Streptococcaceae* and *Leuconostocaceae* were enriched in FNG rather than in FPG. At the genus level, the three most abundant genera were *Faecalibacterium, Clostridium_g4,* and *Bacteroides*, which accounted for an average of 30.5% of sequences (Table 2). The population levels of *Clostridiales_uc_g, Oscillibacter, Ruminococcus, Holdemania,* and *Sutterella* in FPG were significantly higher than in FNG, but that of *Leuconostoc* in FPG was lower than in FNG. Furthermore, the population levels of *Bacteroides* and *Bifidobacterium,* were dramatically increased in FPG whereas those of *Clostridium_g4, Catenibacterium, Eubacterium_g9,* and *Haemophilus* were dramatically increased in FNG. Interestingly, in our previous work, we have found that *Bacteroides* and *Bifidobacterium* could metabolize ginsenoside Rb1 to compound K [16].

**Table 2:** The difference between FPG and FNG in the composition (percent of total sequences) of fecal bacterial genera.

Genus		Compositiona) (%)		
	Total	FPG	FNG	P value
*Faecalibacterium*	15.7±14.75	14.58±18.24	16.84±12.39	0.960
*Clostridium_g4*	8.27±15.39	0.71±0.96	15.83±19.72	0.175
*Bacteroides*	6.50±13.66	14.17±18.87	0.37±0.63	0.077
*Catenibacterium*	6.06±16.04	0.33±0.70	11.80±22.27	0.331
*Roseburia*	4.52±3.96	4.81±4.86	4.27±3.40	0.401
*Ruminococcaceae_uc*	2.92±2.83	4.19±3.46	1.65±1.42	0.222
*Eubacterium_g9*	2.75±4.68	0.73±1.02	4.78±6.17	0.204
*Haemophilus*	2.66±7.37	0.01±0.02	5.96±10.89	0.316
*Prevotella*	2.43±2.37	3.02±2.71	1.84±2.11	0.744
*Clostridium*	2.35±2.92	1.52±2.44	3.17±3.39	0.157
*Dorea*	2.04±1.66	1.19±1.22	2.89±1.70	0.066
*Bifidobacterium*	1.72±5.20	3.31±7.37	0.01±0.01	0.291
*Clostridiales_uc_g*	1.27±1.14	2.08±1.12	0.46±0.08	0.030
*Ruminococcus*	0.82±0.98	1.34±1.19	0.30±0.19	0.046
*Oscillibacter*	0.11±0.15	0.22±0.14	0.006±0.01	0.022
*Leuconostoc*	0.07±0.10	0.004±0.005	0.133±0.104	0.043
*Holdemania*	0.03±0.04	0.052±0.047	ndb)	0.021
*Sutterella*	0.01±0.02	0.026±0.019	0.001±0.003	0.006

a) Mean ±SD (n = 5).

b) not detected. FPG, fecal activity potently metabolizing ginsenoside Rb1 to compound K; FNG, fecal activity non-metabolizing ginsenoside Rb1 to compound K.

**Figure 4:**
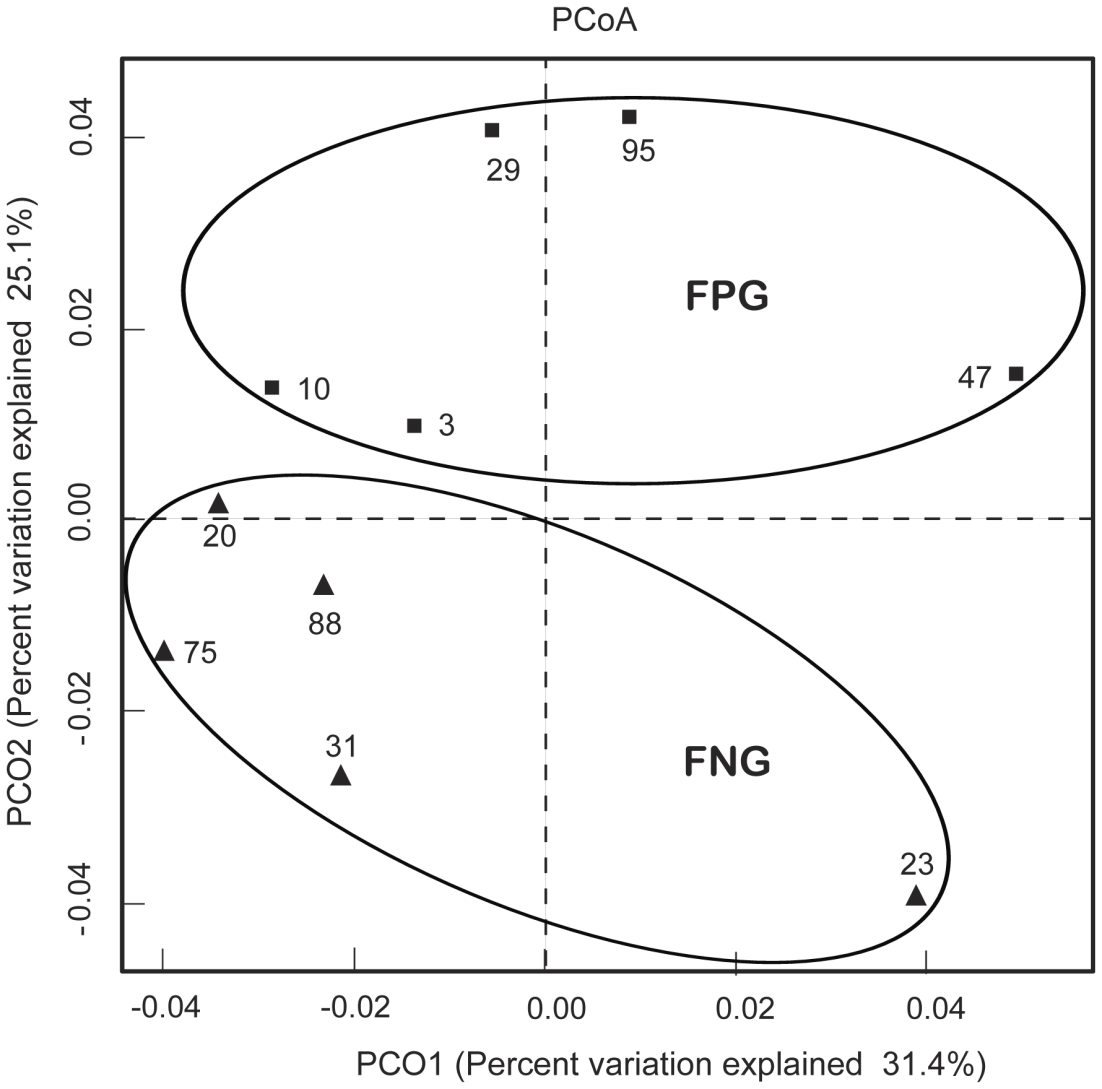
Principal coordinate analysis (PCoA) plot. The plot showed the clustering pattern between FPG and FNG based on weighted pairwise Fast UniFrac analysis. FPG, fecal activity potently metabolizing ginsenoside Rb1 to compound K; FNG, fecal activity non-metabolizing ginsenoside Rb1 to compound K.

We also processed all these sequences at the same length and position to match the length and position of the gut microbiota 16S rRNA gene sequences, computed all pair-wise distances between FPG and FNG and performed Principal Coordinate Analysis (PCoA) to cluster these communities along axes of maximal variance (Fig. 4D). Gut microbial community of each group member was clustered and the maximum variations were 31.4% (PCO1) and 25.1% (PCO2). Furthermore, the gut microbiota compositions of FPG and FNG were segregated on PCO2 by Principal Coordinate Analysis. The difference may be related to gut bacteria metabolizing ginsenoside Rb1, suggesting that diet style, as well as host genetics may affect in molding gut microbiota.

Based on these findings, the difference of the pharmacological effects of orally administrated TCM and its constituents between individuals may be dependent on the composition of gut microbiota.
